# The American Dental Association

**Published:** 1894-11

**Authors:** J. M. Walker


### The American Dental Association.

Thirty-fourth Annual Session, at Old Point Comfort, Va., August 7th, 1894.

Reported for the DENTAL REGISTER by Mrs. J. M. Walker.

[*Continued from October Number, page* 506.]

### NOMENCLATURE.

DR. S. H. GUILFORD, Secretary of Section II, read a report on "Nomenclature," of which an abstract follows:

He said: This subject had never received the attention which its importance demands. While we claim that our profession combines both science and art, yet we lack the most essential requisite of a science, viz: Exactness of expression. The whole literature of dentistry teems with examples of words used interchangeably, which vary greatly in shades of meaning, while others not infrequently fail to convey the meaning intended.

We do not find this so in any other science. In the so-called exact sciences—mathematics, chemistry, astronomy, as well as engineering, navigation—etc., each part, factor, manifestation or act has its single and appropriate designation. Were this not so, confusion would result, sometimes disastrously. In our haste and anxiety to further the material interests of our profession, we have, in a large measure, overlooked the character of the foundations on which we have been building. We can never lay just claim to being scientific until we have a vocabulary suited to our needs, and which will enable us to express our ideas correctly and without danger of misunderstanding or confusion. Reports, more or less satisfactory, on this subject have been presented to our societies from time to time ; individuals, members of the profession, have also defined a nomenclature of their own and offered it for adoption. If the college faculties would all adopt a uniform system and teach it in the schools, good results would follow. But each system offered differs from all others, with individual peculiarities, which, instead of simplifying and systematizing our terminology, only add to the confusion.

In view of these facts, and the unquestionable importance of the subject, the section recommends that steps be taken by the Association to have prepared an intelligible, correct and systematic nomenclature, which by its inherent merits shall commend itself to the profession in general, and be universally adopted. The section was of the opinion that this end can be attained by appointing special committees selected from the men especially interested in each department, whose duty it shall be to carefully and studiously prepare a terminology, accurate and yet simple enough to be readily comprehended. The work would be arduous and long, but the results would be of incalculable value. The section also recommended that the system of dental notation given to the profession by Dr. Corydon Palmer some years ago, consisting in the use of Arabic numerals to indicate the permanent teeth and Roman numbers for the deciduous teeth, combined with horizontal and perpendicular lines to specify the arch and the side of the median line, be adopted by the Association.

On motion of Dr. B. Holly Smith, a permanent committee on terminology, as recommended by Section II., was appointed, as follows :

Drs. S. H. Guilford, L. Ottofy, M. L. Rhein, Thos. Weeks, C. L. Goddard, L. D. Shepard, Grant Molyneaux, D. R. Stubblefield and A. H. Thompson. This committee is expected to report progress annually, and be continued from year to year. The adoption of the system of notation of Dr. Corydon Palmer was referred to this committee.

Dr. Guilford also read a paper by Dr. M. L. Rhein, entitled: "The Etiological Classification of Pyorrhœa Alveolaris."

He said: Various forms of pericemental inflammation with purulent discharge at the gingival border have afforded points of dispute for years. The etiology, pathology and treatment of this disorder will furnish a field for *battle royal* at our meetings until science shall have vanquished this destroyer of dental organs. The names given to this disease are as varied as its manifestations.

The name "Riggs' Disease" was bestowed upon it in honor of the man who first called the attention of the profession to the feasibility of combating its ravages.

The name *Pyorrhœa Alveolaris* has a strong foothold, and it would be as easy to eliminate the word *dyspepsia* from the menclature of the medical profession as *Pyorrhœa Alveolaris* from that of dentistry. But although this name only expresses the single clinical feature of a flow of pus from the alveolus at the gingival border, it has been used indiscriminately to express the most severe and complicated form of the disease in question, as well as the simple and uncomplicated To obviate this difficulty would be to remove one source of contention, and leave time for the investigation of more important matters.

Dr. Rhein submitted a plan of etiological classification of various phases of the disease, retaining the name Pyorrhœa Alveolaris as generally descriptive of any condition where pus flows from the alveolar borders, but classifying the various forms of the disease. He would first make the two grand divisions of:

I. Pyorrhœa Simplex, and II. Pyorrhœa Complex.

I. *Pyorrhœa Simplex* would include all cases of purely local origin and cured by local means. Lack of hygienic conditions, unsanitary conditions are at times the sole cause of a purulent discharge. This is purely local and yields to local treatment. In other cases, malnutrition—a poverty of the life-endowing corpuscles—plays an important role.

II. *Pyorrhœa Complex*, by different writers, called "true pyorrhœa," "phagedenic pericementitis," "hæmatogenic calcic pericementitis," presenting numerous peculiar or obscure clinical features.

To the numerous subdivisions of this grand division, with its complex varieties, Dr. Rhein proposes to add a modifying word, which shall indicate the etiology of each case after careful diagnosis.

Class A would include cases complicated with or due to gout, diabetes, chronic rheumatism, Bright's disease, scurvy, chlorosis, anæmia, leucæmia, pregnancy.

B. Cases having their inception during attacks of acute or infectious diseases, as typhoid fever, tuberculosis, malaria, acute rheumatism, pleurisy, syphilis, etc.

C. Nervous diseases, as spinal diseases, neurasthenia, hysteria.

D. From the toxic effects of drugs, as mercury, lead, iodine.

E. Pyorrhœa sequentia, arising after the prime cause of disease has been removed.

For each of these classes a compound term, such as "gouty pyorrhœa," "mercurial pyorrhœa," "tuberculous pyorrhœa," etc., would be clearly understood, indicating the pathology and treatment of the case in a concise manner without the interjection of any irrelevant matter.

The report of Section VI. included a review of the recent journalistic literature of Pyorrhœa Alveolaris, notably, the writings of Dr. C. N. Peirce, G. V. Black and J. E. Cravens.

Prof. Peirce being requested to open the discussion, said that Dr. Rhein's paper had opened up the subject in a practical manner, and was worthy of notice as an intelligent and interesting effort to differentiate the multiform phases of the group of disorders indiscriminately known as pyorrhœa alveolaris, some of which are amenable to local treatment, while others are not even modified by it. The first step in the treatment is to differentiate the conditions. What has been called "true pyorrhœa," which causes the loss of the teeth, is of constitutional origin, deposits beginning at or near the apical end of the root. The "pockets" are secondary effects. Dr. Black has failed to recognize these conditions, or has not met with them in his experience. He states that ordinary gouty conditions are not attended by the suppurative conditions. It is true this is not usually the case, but numerous instances are on record where suppuration has followed gouty deposits in the joints. The tissues around the root of the tooth are very susceptible to irritation, and have inflammatory conditions and suppuration follow these "serumal" deposits, beginning at or near the apex of the root.

Dr. Abbott: I am a good deal like Dr. Black, in that I have not yet seen the conditions described by Dr. Peirce, with the deposits beginning at the end of the root, and followed by suppuration. I cannot conceive how this can exist without the formation of an abscess and terrific pain and distress before the outpour, the tissues are so dense and unyielding. I believe this disease is due to both local causes and to constitutional disturbances localized in the mouth. I think there is always some local disturbance at the margins of the gum, very slight perhaps, the impaction of particles of food affording a menstruum for the infiltration of lime salts. If the physiological functions of the system were all perfect there would be no such disease, but science says there is no such thing as absolutely perfect physiological action. There is no such thing as an absolutely perfect eye or ear. None of our organs are absolutely perfect in function. Hence the tendency to abnormal deposits of lime salts in the mouth and elsewhere in an effort to get them out of the body in that way. In all this talk about nomenclature and terminology, there is too much "fuss and feathers." What we need is an understanding of principles rather than an accumulation and multiplication of names for the same conditions. Words should be used to express ideas. The simple term pyorrhœa alveolaris expresses clearly the conditions we find.

Dr. Crawford: I will state it differently. The condition known as pyorrhœa alveolaris will never occur in any case without the precedent condition of an issue. Whether of local or constitutional origin, after the removal of *all the teeth* you will never see pyorrhœa alveolaris. Deposits on the roots are the sequellæ of a sinus. The characteristic pockets and all the variable conditions will invariably be found to have had the precedent condition of an issue, though very mild perhaps. Practically, we make more mistakes in physical diagnosis than in anything else. I would not enlarge our vocabulary at the expense of the truth, but I am pleased with Dr. Rhein's division of simple and complicated pyorrhœa, though I doubt the use of the terms "simplex," "complex," "sequellæ," etc., make it any more easily understood than plain language, which we are all competent to understand.

Dr. Abbott: I wish to ask Dr. Crawford what he understands by a "precedent issue?"

Dr. Crawford: A wound—an opening through which infection enters—whether it is a pin point puncture or an erosion as big as your hand. An "issue" is an opening. Mercury manifests itself in ptyalism only where local precedent conditions were favorable. With corresponding precedent pathological conditions elsewhere, it manifests itself in other portions of the body.

Dr. Rhein: I have been studying this disease for twelve years, and my observations lead me to believe firmly in the correctness of the views expressed in my paper. It is true that others, with equally good powers of observation, have reached opposite views; but we are not all equally careful in our observations, we are not all under the same conditions, we do not all meet with the same features. The conditions exist in very variable degrees. We cannot doubt that the conditions as seen by and described by Dr. Peirce do exist, and that he has found these apical deposits without any perceptible break at the gingival border. Because another man has not seen it is no proof that it does not exist. To my mind nothing can account for the varying phases and clinical features presented, except the varying causes found in varying constitutional conditions prevading the system at large. The question as to why these disturbances manifest themselves about the teeth was most nearly answered by the late Dr. Atkinson, when he said there was no tissue in the body so nearly resembling original protoplasm as the mucous membrane about the teeth. The tissues of the socket of a tooth are different from all joint sockets, being the softest and most vulnerable. That is why it is so extremely susceptible to lack of nourishment with consequent retrogression to primeval order.

Dr. John S. Marshall referred to a paper which he had read some years ago before the Medical Association at Washington, on the gouty and rheumatic diathesis, in its relation to the peridental membrane, and spoke of uric acid deposits found at the apex of the root, when the most careful examination with the finest steel probe failed to show any break at the gum margin, or any opening until one was made with the bistoury. A sufficient number of cases of this kind have been found to establish the fact that it does occur. This deposit is a urate of soda. Because this uric acid reaction from serumal calculus, is not found in salivary calculus is no proof of its non-existence. It is found in more cases where there is no history of gout, and where the urine fails to afford the uric acid tests. This disease of the peridental membrane is sometimes the very first premonitory symptom of the rheumatic diathesis. The treatment as per gouty diathesis—cutting off all meat and wine—putting on lithia or distilled water (and I have found distilled water better than lithia in some cases.) As long as that line is followed there are no exacerbations of the disease, but drop it, and it comes back again. One good dinner, with game, champagne, etc., will undo the work of weeks in treating this disease. So long as the patient denies himself he is comparatively free from trouble. So I maintain that these uniform results are proof that the disease is a manifestation of the uric acid diathesis. There is also a condition of pyorrhea alveolaris, resulting from displacements of the uterus and pregnancy. A number of cases have been reported of beneficial results in the mouth from the replacement of the uterus. There are many cases the result of neurasthenic condition, and it will not do to assert dogmatically that it is always due to local conditions.

Dr. Crouse: That there are cases in which the deposits on the root, before there is any external break in the tissues, is beyond a doubt. The correctness of such a diagnosis has been proved by the extraction of the offending tooth.

On the Sunday before Dr. M. S. Dean's death, I dined with him. The day before, "a great big fellow" had compelled me to extract a tooth which presented no signs of anything as the source of trouble, but he had the toothache, and the tooth was sore, and out it must come. It was an upper molar, and on one of the roots I found a tiny spot, black as jet, near where the pulp passed in. I showed it to Dr. Dean and asked how that calculus could have gotten there. The tooth was not at all loose, and gave no evidence externally of being in any way affected. Two or three years later the man came back with the other sixth year molar affected in the same way. In some cases where I have suspected this to be the cause of trouble, it has got better. Either the deposit has been tolerated by nature, or it has perhaps been absorbed and carried off. It is the most distressing disease in dental pathology, and it prevents me from crowning many teeth. We can get rid of every other trouble, but this is incurable.

Dr. Noble said that two years of careful observation had led him to a change of opinion, and he now believes that although it is rare, yet cases do occur with patients of gouty diathesis, of little deposits of serumal calculus near the end of the root with no opening at the margin of the gum. But probably the majority of cases of pyorrhœa alveolaris result from pockets—an "issue," to use Dr. Crawford's term. Very careful observation, however, will lead to the conclusion that Professor Peirce's position is correct.

Dr. W. H. Morgan: It may be so, but I have never yet met with a case where I did not find an opening, either along the alveolus or through the root canal. I have examined very carefully, because these cases give so much trouble. I must repudiate the idea given forth by one speaker, that calculus is ever broken down and absorbed. If in the liver, or bladder, or soft tissues, it may wander around, but it is never absorbed. Nature has three modes of getting rid of foreign bodies, and one of them is by absorption, but we have no evidence that calculi in any part of the body have ever been absorbed.

Dr. Crouse: In one case four upper molars were successively attacked with the same symptoms. I removed the first one to relieve acute suffering, and found a small amount of black deposit on the root. I took out the others as they presented without waiting for protracted acute suffering, though the teeth were perfectly firm, and I found this same black deposit on each one of the four upper molars in the same mouth.

Dr. Frank Abbott: I think this was a case simply of hypnostosis, mistaken for deposits of the other character—an enlargement of the cementum causing periosteal inflammation.

Dr. C. N. Peirce: The evidence is very complete that there is this connection between pyorrhea alveolaris and the gouty diathesis. Every clinician who writes on gout states that as an accompaniment, "the teeth loosen and fall out." Medical practitioners often unwittingly effect a cure of this disease by their anti-gout treatment of malarial conditions. The patient, with his other symptoms, suffers from intense pain at the roots of the teeth, with inflammation of the surrounding tissues.

Abstention from meat and other anti-gout treatment cures this. One case only: a gentleman came to me last January to have his tooth out—was suffering so that he could not stand it any longer—had to walk the floor all night, etc. On examination I found the ravages of pyorrhea, pockets and so on. I asked him what he had been doing for it. He replied, that he was under a physician's care for kidney trouble. I said that if he placed himself in my care I would put him on the dietary and remedial treatment for gout, he assented and was literally cured by this constitutional treatment. I gave the necessary local treatment at the same time, but he had had that for five years elsewhere without benefit. The anti-gout constitutional treatment in connection with local treatment is successful in so many cases that it is only fair to assume the connection between gout and pyorrhœa alveolaris.

Dr. Rhein: The case described by Dr. Crouse represents a number of cases that have been under my observation. Dr. Abbott questions the correctness of Dr. Crouse's diagnosis, but men often assume that other men are not capable of diagnosing obscure cases. For many years the practice has been advocated of removing the pulps in such cases, but in regard to the ultimate result it cannot always be depended upon.

Dr. Taft: In view of the great importance attaching to this subject, I would suggest the appointment of a committee or commission for the special investigation of this subject, to make a report at the next meeting of this Association. If this is done and special work devoted to it, we will get at something more definite than is now known.

One factor has been overlooked in the present discussion, and that is the non-use of the teeth. In many cases defective nutrition is a factor. When one or more teeth are lost the opposing teeth elongate. How is that caused? The teeth do not grow. There is a thickening process going on in the socket, which fills up, and the tooth is gradually thrown out, a thickening of the pericementum, showing that a change is going on in this membrane that is about out of use from lack of normal functions. It is not at all analagous to alveolar abscess; the latter is rapid, with acute inflammation, with loss of structure of all the tissues, both hard and soft. In pyorrhœa alveolaris that is not true; this affection seems confined to the pericemental structure. An ordinary small instrument shows no loss of substance; there is no large cavity about the end of the root in the pericemental membrane involved. What has non-use to do with this? Evidently a change takes place in the pericemental structure when the teeth are out of use. Proper use is necessary for the proper function of this tissue. Take any joint and maintain it in a condition of quiescence—quite free from movement, and it will not be long before there is a changed condition—a filling in or precipitation of calcareous or other matters precipitated from the fluids of the body. In the main, pyorrhœa is a local affection, but systemic influences modify its progress. Some teeth will rest for years without elongating, but others elongate very rapidly, and it is always those that are not used; those that are the most used are the most healthy. There are other points that would make this discussion too long. I move that a committee be appointed to make special investigations along this line.

Dr. John S. Marshall: It is not always easy to decide if an affected tooth has a live or a dead pulp, but there is one positive method, and that is the Faradic current. Applied to a tooth, if the pulp is living you get an instant response. Give the patient one pole and hold the other yourself; place your finger on a known dead tooth and there is no response; place it on a live tooth and the patient jumps; then place it on the suspected tooth, and the Faradic current will tell you positively whether the pulp is alive or dead.

Dr. Bogue: I am heartily in favor of the committee proposed by Dr. Taft, and Dr. Marshall for one of that committee. There are three causes for roughening of the end of the root; the first is from deposits at the apex; second, from absorption, and third, from causes beginning at the gingival margin. A case of the second class lately came under my observation. After treating in vain for six months, I extracted the tooth and found considerable absorption, but no deposits and no discoloration. In three or four places there were points as sharp as needles.

Dr. W. H. Morgan: How would you differentiate whether it was a case of absorption or of malformation? Was the end of the root covered with periosteum?

Dr. Bogue: The patient had been in my hands for many years, and had lost many teeth from salivary calculus in spite of all my efforts. This left lateral was loose, and useless compared with the other teeth, and I saw what I believed to be clearly *absorption* as described.

Dr. Harlan: With reference to deposits on the roots of living teeth, where there is absolutely no opening at the gingival margin of the alveolus, there is no evidence that those deposits originated while the patient was suffering from gout or rheumatism; there is nothing to show that there had not been some antecedent injury sufficient to produce deposits on the root through the agency of micro-organisms. As far as I know, no analysis has been made of deposits on roots of teeth where the continuity of the gingival margins was not destroyed. How many cases of such deposits have been found? Has the difference been established between the deposits on the roots where there is, and where there is not a break in the continuity of the gingival margins? Deposits do occur, and have been found, but I seriously question if it has been demonstrated that the deposits result from the gouty or rheumatic diathesis. Without the formation of a nidus of some character, under the periosteum or on the cementum itself, accessible to bacteria, such deposits cannot take place. It is impossible to produce suppuration without the presence of micro-organisms. As far as I know, micro-organisms of gout and rheumatism have not been discovered in connection with the deposits of this disease.

DR. TAFT: The deposits in question do not depend upon the conditions described by the last speaker. These salts are held in solution in plasma, perhaps in serum, and are deposited in the root from some change taking place, causing precipitation. The solvent is no longer able to hold them in liquid form, and precipitation takes place. I hope we will get men who will work in dead earnest on this commission; push the work hard, and tell us more than has yet been elucidated.

DR. RHEIN: I feel convinced that the prime necessary condition of all the complex varieties of pyorrhœa is mal-nutrition—atrophy. Dr. Harlan says that the claims made have not been clearly demonstrated, but it would take too much time here to detail the causes which have furnished the evidence. It is not implied that gouty conditions are necessary to bring about every case of deposits. They are just as possible in neurasthenia, Bright's disease, diabetes, or from mal-nutrition. There are other forces as well as gout. Toning up the system always has beneficial results.

Subject passed.

At a later session, in accordance with the resolution offered by Dr. Taft, a committee of five, consisting of Drs. C. N. Peirce, John S. Marshall, E. C. Kirk, M. L. Rhein, and J. Taft was appointed as a commission, whose work it shall be to make special investigation in the endeavor to ascertain the etiology, pathology and treatment of pyorrhœa alveolaris, being authorized to draw on the Treasurer for a sum not to exceed $75.00, to cover the necessary expense of this work.

### SECTION VI. PHYSIOLOGY AND ETIOLOGY.

DR. S. B. PALMER, Syracuse, N. Y., read a paper on the "Etiology of Dental Caries."

Dr. Palmer referred to papers read by him twenty years ago, upon the chemical and galvanic action between tooth substance and the materials used for their preservation. His teachings on this subject were in advance of the times and met with much opposition, but during the twenty years that have passed since those views were advanced, science has overcome prejudice and the principles then advanced are quite generally admitted. Dr. Palmer proceeded to give a summary of the conclusions reached, taking the filling materials as the elements of a battery, defining principles and citing cases. In regard to the potential relations of matter, electrolysis must be understood as the principle governing the composition and decomposition of matter. Following this to its terminus in the oral cavity, dental caries is an effect of the electro-potential relations of matter. Dr. Palmer then defined the terms used in electrical science, describing the phenomenon known as electrolysis with its application to dental science, a tooth being considered as an electrolyte. The electro-chemical theory is founded on natural laws. There is a continuity of law through the inorganic, the vegetable and the animal. Organized tissues are subject to decomposition and decomposition resulting from electro-chemical action.

When gold is malletted against the frail walls of a tooth the bruised surface of porous dentine becomes an electrolyte. The metal used being at a potential above that of the dentine, decay results as an effect of electrolysis. This is entirely apart from primary and independent of the caries arising from ferments, organisms and organic acids. Very important discoveries have been made in regard to chemical action in the mouth and the use of antiseptics to prevent fermentation and destroying organisms. But this does not offer a remedy for the arrest of decay from the action of oxides.

Dr. Palmer then described the peculiar complications found in the oral cavity, with its undercurrent of oxygen—a negative element—the tooth an electrolyte raised to the animal or vegetable plane ready to be acted upon and soon destroyed. If the bruised portion of an apple is cut out with a sharp blade and the surface dried the wound heals. It is the same with a tooth; cut out the disorganized portion, insert the filling of such material as shall exclude moisture and oxygen and there will be no secondary decay. If the dentine is below normal, supply the deficiency by an agent not affected by oxygen; as the ranchman burns a space around his stakes and satisfies the demands of oxygen so that fire stops at the line prescribed. If softened dentine and cavities in deciduous teeth are treated with nitrate of silver the oxide of silver raises the potential of the dentine above that of the secretions of the mouth and caries is arrested or prevented. Nineteen years ago Dr. Palmer wrote "on this principle nitrate of silver turns the teeth dark and checks caries." Oxygen under the laws which govern the positive and negative relations of the elements is the primary agent which produces dental caries, and to arrest decay we utilize the oxides—which are proof against oxygen—through the means of metal fillings which are of higher potential than the organic constituents of dentine. Tin fillings furnish an insoluble stannic oxide through the simple process of oxidation. No decay occurs as long as the black carbonate remains, but it is soft and does not resist the brush or pick. If gold and tin are joined in a filling, by electrolytic action, there is an interchange of atoms and an alloy of tin and gold is formed, raising, by induction, a portion of the tin to the same potential as gold, but if not more than two layers of tin are used, the dentine is furnished with a carbonate and gold can be successfully used where otherwise it would be a failure. Decay around gold fillings is in obedience to laws which manipulative ability cannot counteract. The current from the filling of higher potential decomposes moisture in the dentine, liberating oxygen, which is attended with an acid, dissolution of lime salts being the result.

In all alloyed amalgams there are unamalgamated particles in the mass which are negative, the amalgamated portion being positive, consequently local galvanic action is established, oxidation filling normal dentine with oxides and sulphide and no harm is done; *but* if the dentine is below normal, local action is too vigorous, the lime element of the dentine is dissolved, the cavity enlarged and the plug reduced. Line the cavity in such teeth with tin, pressing it in with a ball burnisher and you get the benefits of oxidation filling the tubuli with an insoluble compound, while no local action takes place to cause shrinkage of the plug. The stannic oxide is not objectionable in color and prevents shrinkage from galvanic action which occurs without the tin lining. Suggestions for root filling are based on the same principles. All fillings below the gingival margin are in an alkaline field. Here oxygen gives an alkaline reaction which is the reason why unfilled root-canals do not enlarge in the same proportion as cavities in tooth crowns. Here acid treatment is indicated and is beneficial in destroying micro-organisms. In filling root-canals the opening at the apex is a spring which cannot be dried up; capillary attraction keeps the fine canals filled until the leak is stopped. If an oily substance is used some oil remians which cannot be volatilized even with broaches heated with electricity. In time the oil is oxidized and the canal becomes filled with septic matter. But introduce the liquid of oxychloride, which checks bleeding and fill with a thin mix; force in a gutta-percha point which forces all excess of fluid out through the apex and the opening is closed. The permanence of gutta-percha consists in the oxide which remains after the gum is oxidized; this preponderance of oxide prevents further oxidation. The liquid phosphate is not metallic, hence the benefits of the oxychloride over the oxyphosphate. Dr. Palmer described minutely the laboratory experiments which confirm the positions taken and the principles established. In conclusion he said: Dental caries is an effect of universal principle, based upon the potential relations of matter, oxygen being the principal element aided by electrolysis and capillary attraction under the direction of electrical energy.

[*To be continued.*]

